# Distance-Based Opportunistic Mobile Data Offloading

**DOI:** 10.3390/s16060878

**Published:** 2016-06-15

**Authors:** Xiaofeng Lu, Pietro Lio, Pan Hui

**Affiliations:** 1 School of Computer Science, Beijing University of Posts and Telecommunications, Beijing 100876, China; 2 Computer Laboratory, University of Cambridge, Cambridge CB3 0FD, UK; pietro.lio@cl.cam.ac.uk; 3 Department of Computer Science and Engineering, Hong Kong University of Science and Technology, Clear Water Bay, Hong Kong, China; panhui@cse.ust.hk

**Keywords:** Mobile data offloading, delay tolerant, opportunistic communications, content dissemination model

## Abstract

Cellular network data traffic can be offload onto opportunistic networks. This paper proposes a Distance-based Opportunistic Publish/Subscribe (DOPS) content dissemination model, which is composed of three layers: application layer, decision-making layer and network layer. When a user wants new content, he/she subscribes on a subscribing server. Users having the contents decide whether to deliver the contents to the subscriber based on the distance information. If in the meantime a content owner has traveled further in the immediate past time than the distance between the owner and the subscriber, the content owner will send the content to the subscriber through opportunistic routing. Simulations provide an evaluation of the data traffic offloading efficiency of DOPS.

## 1. Introduction

As smartphones have become more popular, more people are reading news and watching videos on those portable devices than before. Cisco reported that mobile data traffic increased 74% in 2015 [1]. In 2015, 51% of total mobile data was offloaded onto fixed networks, such as WiFi or femtocell. The most popular scenario of offloading mobile data through WiFi is when a user’s smartphone contacts a WiFi router located in his/her office or home. However, when people are outside of their offices and houses, they have to access Internet through a cellular network if there are no WiFi or femtocell infrastructures.

Recently, researchers have being studying how to offload mobile data from cellular networks onto opportunistic networks [2,3,4,5,6,7,8,9,10,11,12,13,14,15]. In opportunistic networks, it is difficult to build a continuous end-to-end routing path, so the networks are disruption tolerant. In such networks, a message cannot be directly routed to its destination from the source. The message is forwarded in a store-and-forward method. The source of a message sends its messages to some encountered nodes and these nodes forward the messages to some new nodes when they meet other nodes at some other places [2]. In this method, there would be many copies of one message, and if one copy is finally received by the destination, then the message is delivered successfully.

The delivery delay of opportunistic routing is longer than that of a fixed WiFi network because of its intermittent connectivities. However, people find that for some applications they can afford some delivery delay, such as when downloading movie trailers or multimedia magazines. Thus, these kinds of time insensitive applications or data can be offloaded from cellular networks onto opportunistic networks.

In the study of offloading mobile data from cellular network to opportunistic networks, researchers assume that a node will publish the content or data to opportunistic network if someone subscribes it, whereas we think this principle is not suitable for a large environment. For example, node A in city-1 subscribes to content and node B who is in city-2 has this content. City-1 is far away from city-2. If node B publishes the content onto an opportunistic network in city-2, the probability that this content can be delivered to city-1 and received by node A within a certain limited time is scant. Therefore, we think the distance between the subscriber and the provider of the content impacts the opportunistic routing performance and offloading efficiency.

In this paper we study the opportunistic data offloading from cellular networks through direct wireless communications between smartphones, using WiFi and Bluetooth. The main contributions of this paper are: (1) we study the issue of subscription selection which has not been studied previously; (2) we propose a distance-based mobile data opportunistic offloading model that we have called Distance-based Opportunistic Publish/Subscribe (DOPS).

When a user wants a specific content, he/she sends a content request to a content subscribing server (CSS) through an Internet connection. When another user having the requested content knows that the content is requested, he/she delivers the content to the requestor through opportunistic forwarding if the distance between the two users is shorter than the longest distance it travelled recently during the time duration before the subscription’s deadline. In some cases, many users may want the same content, and the traffic offloading efficiency increases if these subscribers of a common content receive the content through opportunistic networks. DOPS cannot work for time sensitive applications, such as video streaming. The content suitable to be delivered in DOPS is time insensitive data, such as multimedia newspapers, music, and movie trailers. Small video and music files are delivered as bulk files, not streaming files, which means the receiver cannot open the file before receiving it completely.

## 2. Related Work

There are two strategies to offload cellular networks traffic: one is to offload through WiFi access points and another is to offload onto opportunistic networks [3]. In the study of offloading cellular networks traffic through WiFi routers (APs), Dimatteo studied how many WiFi routers were needed to guarantee a certain quality of service [3]. Lee studied the delayed offloading [4]. When a user wants some data, his/her smartphone does not download it from cellular network. It waits to download the data until it can contact a WiFi infrastructure. With tens of minutes delay, 70%–90% of cellular network traffic can be offloaded. Car-Fi offloads data traffic from cellular networks to existing home Wi-Fi routers [5]. This traffic offloading method needs WiFi infrastructures, but the offloading efficiency of this method reduces if the WiFi infrastructure is insufficient [6].

The first study about offloading cellular traffic through opportunistic communication was done by Han *et al.* [7]. They studied how to select the initial k nodes as the target-set selection. Huang *et al.* studied how to extend a publish/subscribe system to a mobile network [8]. Meanwhile, they also assumed that nodes in the mobile network are contacted. However, the mobile network composed of mobile smartphones is intermittently connected networks, not *ad-hoc* networks. Push-and-track offloads traffic through opportunistic networks [9]. In push-and-track, the user sends acknowledgments to the server while receiving the content and the server decides whether to push the content into the network. Sometimes, the number of users is very large, and the server has to handle tens of thousands of subscriptions every day. Then the server will become the bottleneck of the system. In Valerio’s research on offloading with opportunistic networks, if a node cannot get the wanted content from the contacted nodes, it will download it through cellular networks [10]. After a node downloads its requested content, the node sends the content to some random neighbors in the same district. This method does not suit a scenario with low node density. Li *et al.* studied DTN-based mobile data offloading [11]. In their study, a service provider sends the data to some nodes and these nodes send the data to the subscribers with two-hop routing algorithm. Mehmeti *et al.* studied the average delay equation for delayed offloading [12]. Kouyoumdjieva *et al.* studied the relation between the smartphone’s energy and offloading efficiency, and proposed an energy-aware algorithm for opportunistic networks [13]. In addition, some researchers have begun to study the economics of mobile data offloading [14,15]. Anyway, none of these works considers the distance between the subscriber and the node that has the content, but this distance may determine whether the traffic offloading works. Also, these works do not take the deadline of the subscription into consideration, which means the subscription table will soon grow hugely and the subscribing server cannot work efficiently.

## 3. Distance-Based Opportunistic Push/Subscribe Content Dissemination Model

### 3.1. Hybrid Wireless Communication

We assume the smartphones in our study can communicate through cellular networks and WiFi networks, respectively. First of all, a smartphone is able to contact the cellular network base-station to access the Internet. Secondly, the smartphone is able to contact a WiFi router as well. In addition, the smartphone is supposed to be able to make direct WiFi contacts. WiFi direct contact is a new capability that builds a peer-to-peer connection between two WiFi devices. Two smartphones with WiFi direct communication ability can transmit and receive data without a WiFi router [16].

### 3.2. Introduction of DOPS

**Figure 1:**
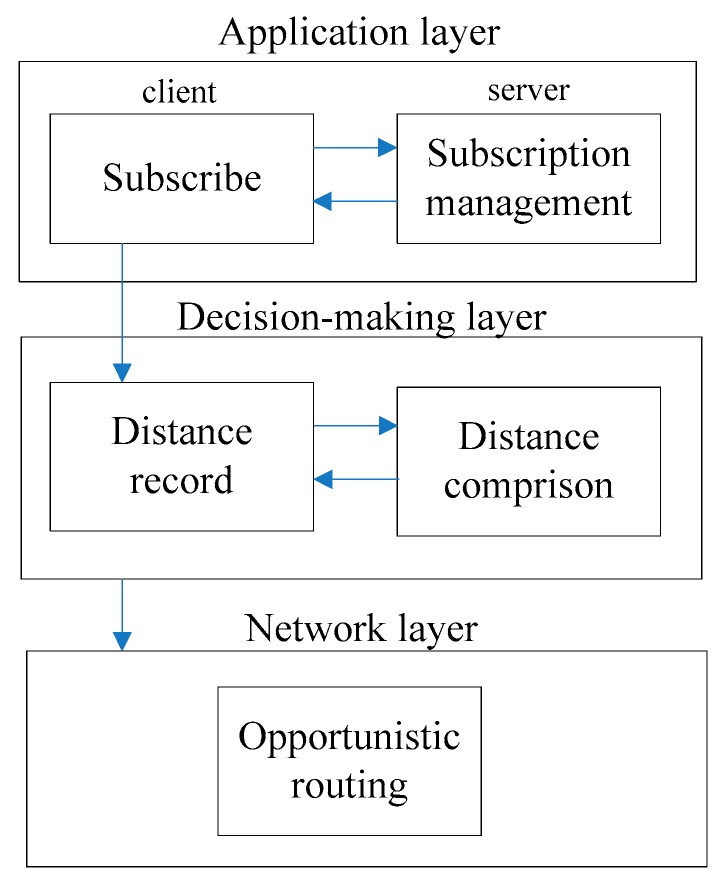
The structure of DOPS.

DOPS content dissemination model we proposed in this paper is composed of three layers: Application layer, Decision-making layer and Network layer. The structure of DOPS is shown in Figure 1.

The application layer copes with the things about the subscribing content and subscription management. The decision-making layer decides whether to publish the content requested by others. The network layer deals with relay nodes selection. Different opportunistic routing protocols can be selected to work in this layer.

### 3.3. Application Layer

#### 3.3.1. Subscribing Content

When a user wants some new content, he/she sends a content request to the content subscribing server. We call the user a subscriber. The content subscribing server adds a subscription to it. The subscription is composed of the name of the content, the user’s ID, the deadline of the subscription and the subscriber’s location. The deadline of a subscription is similar to the time to live (TTL) of a network packet. The user will not download the content through cellular network until the deadline.

If a user subscribes to content, the user’s smartphone gets its current GPS location and records it in the subscription.

#### 3.3.2. Subscription Management

When a user subscribes to some new content, the user sets how many days it can wait for the content. If a user has received the requested content before the deadline, it responds to the CSS and removes its subscription for this content. If a user fails to receive the requested content, the user can extend the deadline. However, if the user does not extend the deadline, CSS deletes the user’s subscription after a certain time. When a user subscribes to the content, the user just sets how many days or hours it is willing to wait from the current time rather than an exact time point. CSS calculates the exact time point of the deadline of this subscription, so it is not necessary that all users’ smartphones clocks be synchronous. All subscriptions are listed according to the sequence of their deadlines. If the deadline of a subscription is very near the current time, the subscription is at the top of the subscription table. If the deadline of a subscription is still many days from now, the subscription is at the bottom of the subscription table. When a provider accesses CSS and check the subscription list, it will check the subscription list from top to bottom.

### 3.4. Decision-Making Layer

#### 3.4.1. Principle of Publishing Content

Among all users, some users may have the requested contents of other users. In this paper, we call the node that has others’ requested content a provider. However, even though a provider has the requested content, it is not obligatory that the provider publish its content. If the provider is very far away from the subscriber, the provider will not publish the requested content because the probability of the subscriber receiving the content is very low.

Let $d_{ps}$ be the distance from the provider to the subscriber, $d_{t}\left(x\right)$ be the distance of the provider travels in past x days. For instance, $d_{t}\left(2\right)$ means the distance of the provider travelled in past 2 days. If a provider accesses CSS and has the subscribed content in one of the subscriptions, the provider publishes the content if Equation (1) is true:

(1) $$d_{ps}\leq Max\left(\left\{d_{t}\left(1\right),d_{t}\left(2\right),\ldots ,d_{t}\left(Rd\right)\right\}\right)$$

**Figure 2:**
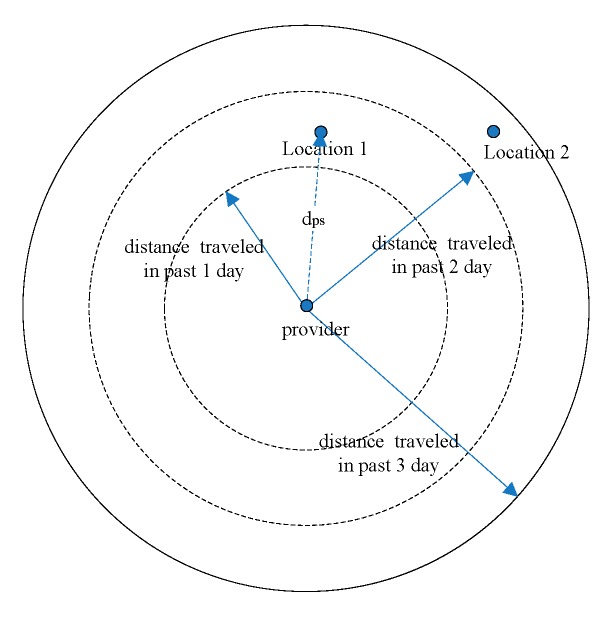
Illustrate of $d_{\text{ps}}$ and $Max\left(\left\{d_{t}\left(1\right)\right\}\right)$, $\;Max\left(\left\{d_{t}\left(1\right),\;d_{t}\left(2\right)\right\}\right)$, $\;Max\left(\left\{d_{t}\left(1\right),\;d_{t}\left(2\right),\;\;d_{t}\left(3\right)\right\}\right)$.

In Equation (1), *Rd* is the remaining days from the current date to the deadline. $Max\left(\left\{d_{t}\left(1\right),d_{t}\left(2\right),\ldots ,d_{t}\left(Rd\right)\right\}\right)$ is the longest distance that the provider has travelled in the past *Rd* days. For example, if the deadline is the day after tomorrow, *Rd* is 2. If the subscriber’s location is at location 1 in Figure 2, the provider will publish the content. If the subscriber’s location is at location 2, the provider will not publish the content.

#### 3.4.2. Publishing Content

Every node periodically records its location. The location file format is (date,$\;GPS_{x},GPS_{y}$). When a provider accesses CSS, it checks the subscription table. If it has the subscribed content in a subscription, the provider gets the deadline of the subscription and the subscriber’s location. Then it calculates the remaining days from the current date to the deadline. For example, if the remaining days are 3 days, it reads the past 3 days’ location records from its location file. Then the provider calculates the distances from its current location to each location ($GPS_{x},GPS_{y}$) in the past 3 days, and it gets the longest distance $d_{max}$. Afterwards, it computes $d_{ps}$, which is the distance from current location to the subscriber’s location. If $d_{max}\geq d_{ps}$, the provider will publish the content, otherwise, it will not.

**Figure 3:**
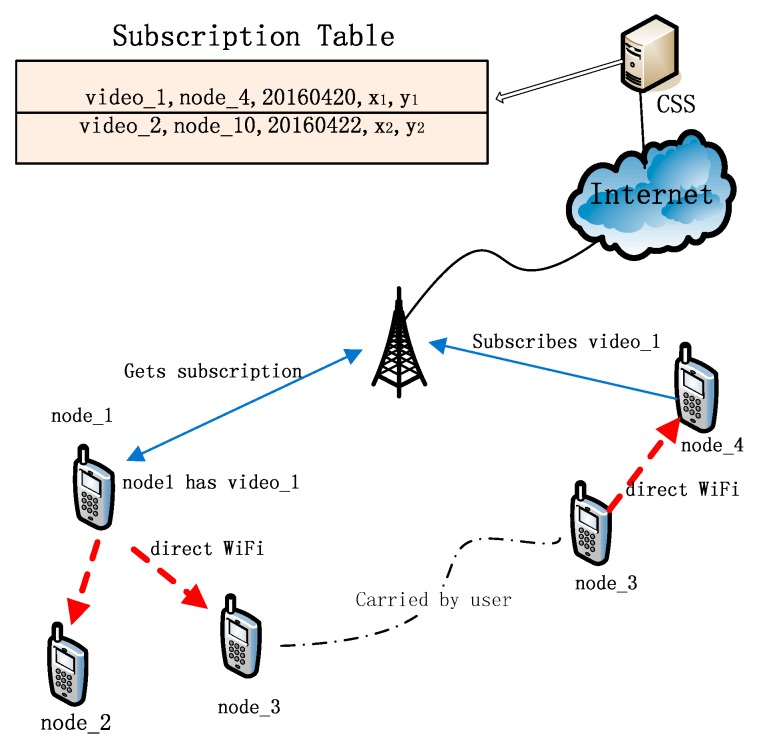
An example of subscribing content and opportunistic publishing content in DOPS.

In Figure 3, node_4 subscribes to video_1 on CSS and node_1 has this video. When node_1 contacts CSS, it finds that a node subscribed video_1. Node_1 gets the deadline of the subscription and the subscriber’s location, (x_1_, y_1_). For example, if today is 19 April 2016, the remaining days from today to 20 April 2016 is 1 day. Node_1 calculates the distance $d_{ps}\;\;$between it and (x_1_, y_1_). Assume $d_{ps}$ to be 4.5 km. Node_1 calculates the longest distance it travelled yesterday. Suppose $d_{max}$ to be 7 kilometers, node_1 begins to publish video_1 onto the opportunistic network through direct WiFi connections because $d_{max}>d_{ps}$. Node_1 sends video_1 to node_2 and node_3. Node_3 is carried by its user from one place to another place and meets node_4. Node_3 transmits video_1 to node_4 when they encounter each other.

### 3.5. Network Layer

The network layer deals with how to select the relay nodes so as to increase the delivery performance [17,18,19,20,21,22,23,24,25]. Selecting the next hop is the responsibility of routing protocols. We do not assign a specific opportunistic routing protocol for DOPS. Many opportunistic routing protocols could be employed here. Literatures [17,18,19,20] are opportunistic routing developed for delay tolerant networks. Literatures [21,22,23] are opportunistic routing protocols specially designed for subscribe/publish. 

DPSP is publish/subscribe-based routing protocol for DTN networks [21]. When a router selects the relay nodes, it considers local resource constraints. Gao *et al.* studied the way of improving the cost-effectiveness of multicast by social analysis [22]. When a node selects relay nodes, it selects the nodes based on social centrality and social community. This approach can reduce the number of relays. MuRIS scheme allows nodes to deliver content via chosen paths, which can reach more subscribers at intermediate hops [23].

### 3.6. Traffic Offloading by Multi-Receiver

Sometimes, several nodes will subscribe to same content. There would be many subscriptions for a common content in CSS with different deadlines. Let *subscriber ratio* be the percent of the subscribers of a common content. While a content is routed to the subscriber, some receivers might be subscribers to this content. Once a node receives the subscribed content, it repeals its subscription for this content. Let *offloading efficiency* represent the ratio of the traffic offloaded from cellular networks to the total traffic includes offloaded traffic and cellular network traffic:

(2) $$offloading\;efficiency=\frac{traffic\;offloaded\;from\;cellular\;networks}{total\;traffic}$$

**Figure 4:**
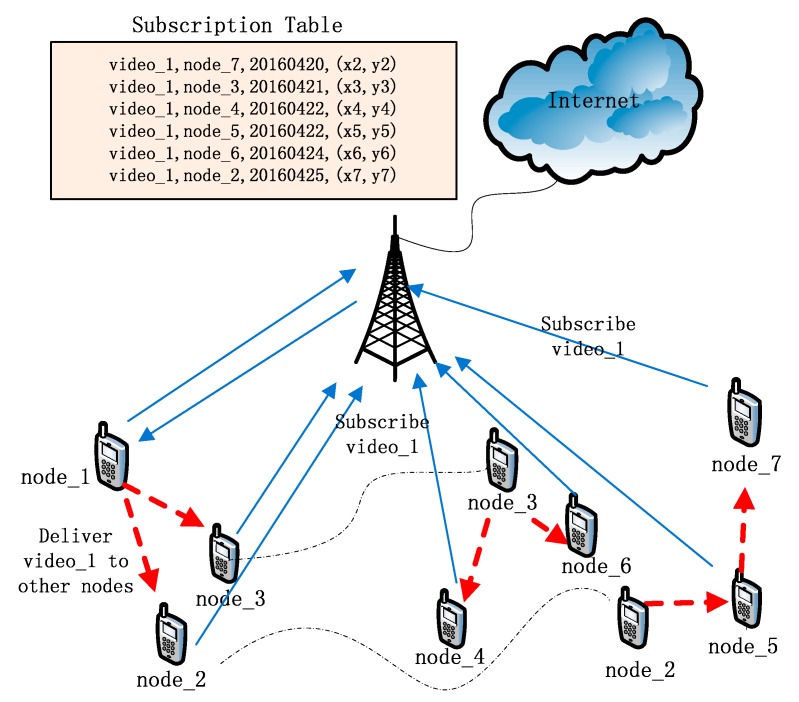
When a relay receives its subscribed content, the relay removes itself from the subscription table.

For example, node_2 to node_7 subscribed to video_1 as Figure 4 shows. Node_1 gets video_1 through a cellular network. Node_1 contacts CSS and knows that node_7 subscribed to video_1 many days ago. After comparing $d_{max}$ and $d_{ps}$, node_1 decides to send video_1 to node_7. When node_1 encounters node_2 and node_3, it sends video_1 through a direct WiFi connection to node_2 and node_3 that also have subscribed to video_1 on CSS. After these two nodes receive video_1, they repeal their subscriptions to video_1 on CSS. However, as they are not the destination of the content delivery, they continue to transmit it to other nodes, and node_2 to node_7 receive video_1 before the deadlines of their subscriptions. In this case, only when node_1 downloaded video_1 through the cellular network, 6/7 cellular network traffic is offloaded onto an opportunistic network and the offloading efficiency is 85.7%.

## 4. Performance Evaluation

### 4.1. Simulation Setup

We evaluate the offloading efficiency of DOPS based on the simulation data from ONE, a simulation software for opportunistic network environments. In the simulator, the nodes were vehicles that traveled along the roads and the mobility model of these nodes was RandomWayPoint. The road map used in the simulation is Helsinki’s city area. The nodes density nearby the main streets was higher than in the suburb. The content was supposed to be music files and those files are from 2 M to 5 MB in size. The parameters we used in the simulation are listed in Table 1. Nodes communicate with each other through WiFi connections. Every 25 to 75 s, a randomly selected node downloaded a new music through the cellular networks. The subscribers were randomly selected from all nodes. The subscriber ratio in each round of simulation was constant. The subscriber ratios were 1%, 2%, …, 10%. We ran the simulation for 10 rounds and the results were the average of the simulation data of 10 rounds. The deadline of all the subscriptions is after 12 h. The opportunistic routing was Maxprop that is provided by the simulator and its delivery performance is very high.

### 4.2. Contact Time

**Figure 5:**
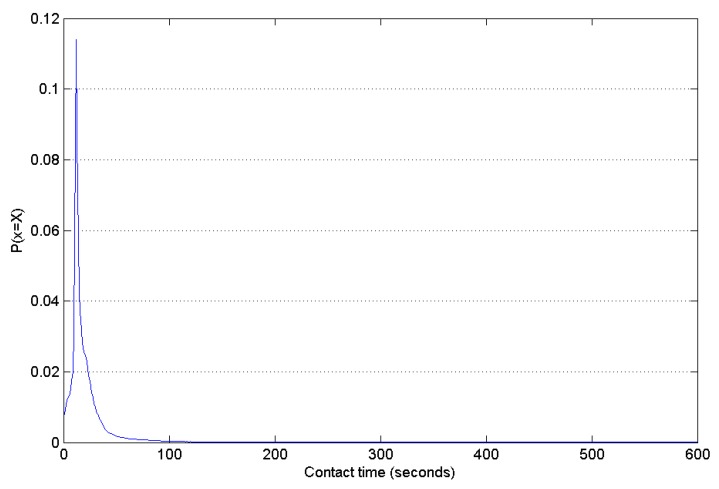
Contact time distribution.

The contact time distribution between nodes indicates how much data can be transmitted and received. In the simulation, the percent of contact time shorter than 10 s is 12.2% and the percent of contact time shorter than 5 s is 5.5%, hence most of contact time is longer than 5 s as Figure 5 shows. Assuming the WiFi bandwidth to be 10 MB/s, if nodes transmit a video no larger than 55 M, the probability of transferring the file successfully among all contacts is more than 94.5%. Let P (contact time ≥ X) be the probability of contact time longer than X s.

### 4.3. Result: Traffic Offloading Efficiency

**Figure 6:**
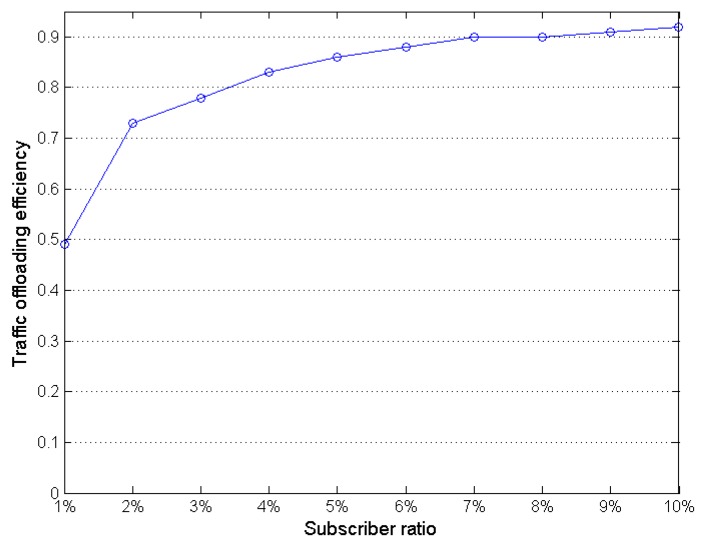
Traffic offloading ratios under different subscriber ratios.

All nodes are supposed to participate in content dissemination. We assume some subscribers would request the same content. The more users are interested in the same content, the higher the offloading efficiency is. In the simulation, the average delivery ratio is 99.6%, which means that 99.6% of all users receive each file. Figure 6 shows the relation between the traffic offloading efficiency and the subscriber ratio. In our study, the total number of users is 180. If two users subscribe to the same music, the subscriber ratio is 1%. Assume one of them got the initial file through a cellular network and another got the file through opportunistic routing, then the offloading efficiency is 50%. If 16 users have subscribed a same music, the subscriber ratio is $\frac{16}{180}\approx 9\%$. Fifteen subscribers among them got the file by opportunistic routing, so the offloading efficiency is 93.75%. As the delivery ratio of the simulation was very high (99.6%), the morphological feature of the curve in Figure 6 approximates the morphological feature of $y=\frac{x}{x+1},\left(x>0\right)$. When the subscriber ratio increases at the beginning phase, the offloading efficiency increases rapidly. When the subscriber ratio is larger than 7%, the growth rate of offloading efficiency slows down.

The deadlines of all subscriptions were after 12 h from the beginning of the simulation. The average delivery delay from when a file was published to the time it was received was 3.5 min. Compared with 12 h, 3.5 min is a short time duration. Hence, the time a subscriber can receive its required content depends on the frequency with which the providers access the CSS and find the subscription. If a smartphone accesses a CSS every 30 min and some smartphones have the subscribed content, then the probability of a subscriber receiving a file smaller than 55 MB within (30 + 3.5) min is higher than P(contact time ≥ 5) × delivery ratio = 94.5% × 99.6% = 94.1%.

**Figure 7:**
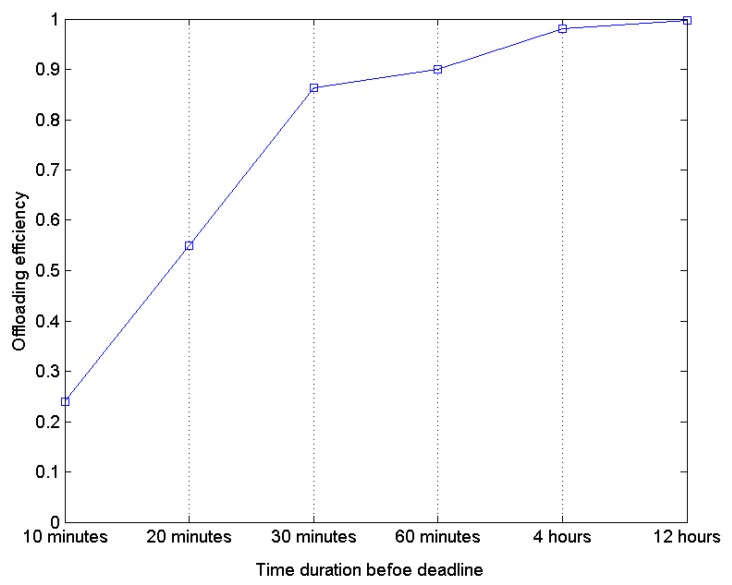
Offloading efficiency with the time before deadline.

The time length before the deadline impacts the offloading efficiency. If a user sets the deadline just 10 min later, it means the user potentially hopes the content provider to be nearby. However, the probability of this situation being true is very low. If the user can wait several hours to get the content, more users would access CSS and the probability that a provider sends the requested content to the subscriber increases, as Figure 7 shows.

If a user downloads a video through a 3G cellular network, the user must pay the bandwidth usage fee. For example, if a video file is 100 MB, it will cost the user 8.3 min to download it through 3G cellular network at an average practical bandwidth of 200 KB/s, and the user has to pay about 1 dollar for this bandwidth usage in China. If a person downloads files through 3G every day, he/she has to pay a lot of money. Therefore, traffic offloading through the opportunistic routing can help subscribers save money.

## 5. Discussion

Some Internet websites, such as youtube.com and youku.com, provide lots of videos every day. If all Internet users watch videos from the websites directly, the websites have to rent a high bandwidth from the cellular network operators and pay a lot of money for this. Therefore, these websites tend to build a peer-to-peer Content Delivery Network CDN composed of mobile smartphones. On one hand, some users can subscribe a content and wait to receive it. Then, these websites can decrease the bandwidth usage fee. On the other hand, if a user can receive the request content through opportunistic routing, the user does not need to pay for the cellular network bandwidth usage fee as well.

## 6. Conclusions

In DOPS, when a user wants some content without downloading it directly through a cellular network, the user subscribes to the required content on a CSS through the cellular network. Before the deadline of the subscription, the user will not download it. Some other users will contact CSS and find the subscriptions. If a user has the content subscribed by others, the user calculates $\;d_{max}$ and $d_{ps}$. If $d_{max}\geq d_{ps}$, the user sends the content to some encountering users by opportunistic routing and the content is delivered through a WiFi direct connection. The simulation results show that DOPS can offload 50%–94.1% of cellular network traffic with the subscriber percentage being 1% to 10%. This technology can be used in Internet content provision, by which the websites can save a lot on cellular network bandwidth fees. For future work, we will further study how to manage the subscriptions more efficiently.

## Figures and Tables

**Table 1:** The parameters in the simulation.

Parameter	Value
Number of nodes	180
TTL	60 min
Wi-Fi transmission range	100 m
Size of the content	2 M~5 M
Simulation time	1 day
Transmission speed	10 Mbps
Simulation area	15.3 km^2^
Nodes travel speed	7 to 10 m/s
Node‘s buffer	100 M
Opportunistic routing	Maxprop
